# Assessment of the Quality of Life and Family Function in Attention Deficit Hyperactivity Disorder Caregivers in Al-Ahsa, Saudi Arabia

**DOI:** 10.7759/cureus.70161

**Published:** 2024-09-25

**Authors:** Jihad Algadeeb, Essa Mohammed AlSaleh, Rahma B AlGadeeb, Huda Abdulaziz S Alkhoufi, Ali Jawad Alsaad

**Affiliations:** 1 Preventive Medicine, Al-Ahsa Health Cluster, Ministry of Health, Al-Ahsa, SAU; 2 Infection Control, Directorate of Health Affairs, Ministry of Health, Al-Ahsa, SAU; 3 Developmental and Behavioral Disorders, Maternity and Children Hospital, Al-Ahsa Health Cluster, Ministry of Health, Al-Ahsa, SAU; 4 Clinical Neuroscience, Faculty of Medicine, King Faisal University, Al Hofuf, SAU

**Keywords:** adhd, caregivers, family burden, family dysfunction, quality of life, saudi arabia

## Abstract

Background

Attention deficit hyperactivity disorder (ADHD) is the most prevalent neurobehavioral disorder in children and teenagers. The condition is debilitating, affecting numerous cognitive and behavioral processes and leading to substantial long-term consequences. Affected individuals, their families, and society as a whole bear a heavy functional, psychological, and financial cost.

Aim

The objective of this study was to evaluate the quality of life (QOL) among parents of children with ADHD diagnoses, as well as the burden and level of dysfunction in the family. Additionally, to ascertain the sociodemographic factors that influence these issues.

Methods

This cross-sectional study used an online survey to collect data from caregivers of children with a confirmed diagnosis of any type of ADHD who were exhibiting symptoms before the age of seven, speaking Arabic fluently and consented to take part in the study. The Arabic version questionnaire of the World Health Organization Quality of Life instrument (WHOQOL-BREF) was used to assess the QOL, and the Zarit Burden Interview questionnaire was employed to assess the degree of the experienced burden. Participants were recruited by a systematic random sampling method.

Results

A total of 103 ADHD caregivers were included in the study. A total of 89 out of 103 (86.4%) participants reported having various degrees of burden. There was a significant relation between the marital status of the caregiver and the degree of burden (p=0.024). Divorced caregivers were (4, 57.1%) severely burdened. Ninety out of 103 participants (87.4%) reported having dysfunctional families, and the majority of them (60, 60.1%) reported moderate family dysfunction. All quality-of-life domains were negatively impacted, with the environmental domain experiencing the most disruption. The overall four subdomains of QOL did not differ significantly with various sociodemographic characteristics.

Conclusion

There was a high prevalence of burden and family dysfunction among caregivers of ADHD children. Marital status was associated with a significant impact on the level of burden. In addition, there was a detrimental influence on every aspect of quality of life, with the environmental domain suffering the most. It is important to take into account family therapy and other interventions that strengthen caregivers' and families' relationships.

## Introduction

Attention deficit hyperactivity disorder (ADHD) is a common childhood behavioral disorder characterized by abnormally high levels of impulsivity, hyperactivity, and inattention, and it usually starts before the age of 12 years [1]. The affected children often have disruptive behavior, dramatic mood swings, poor frustration tolerance, conduct disorder, and one or more learning disabilities [2].

ADHD is the most common neurobehavioral disorder in children and adolescents, with a global prevalence of 7.6% in children aged three to 12 years and 5.6% in adolescents aged 12-18 years [3]. The prevalence of ADHD in Saudi Arabia was recently examined in a systematic review, which documented a pooled prevalence of 12.4% [95% confidence interval (CI): 5.4%-26%] [4]. This is higher than the 10.3% (95% CI: 0.081 to 0.129) reported for the Middle East and North Africa region [5].

The exact etiology of ADHD is unknown, and the onset of ADHD has been connected to a wide range of hereditary and environmental factors. It has also been linked to peripheral and localized inflammation, with an increasing evidence indicating that the mother's inflammatory condition during pregnancy is connected with the diagnosis of ADHD in their children [6]. In Saudi Arabia, specific risk factors have been linked to the increased likelihood of ADHD including experiencing psychological disorders, incapacitating muscle pain symptoms, insufficient vitamin B, or allergic reactions during pregnancy [4].

ADHD is a devastating disease associated with significant long-term effects and impairs many cognitive and behavioral functions, causing a considerable functional, psychological, and economic burden on affected individuals, their families, and society [7]. 

Caring for children with ADHD is a critical responsibility of parents. Their duties include things like starting and assisting with the process of finding expert help, handling the complexities of ADHD treatment, and addressing the significant impact that ADHD has on their children's educational experiences [8]. A systematic review included studies focused on ADHD or autism spectrum disorder children revealing an adverse impact on the psychological well-being and quality of life (QOL) of their parents [9].

Studies highlighting this burden help healthcare professionals take interventions to support parents and improve their well-being as well as provide data for decision-makers to develop appropriate measures to address this problem. Despite this, there is a paucity of research on the influence of ADHD on the quality of life of parents in Saudi Arabia. In the Medina region, Alnakhli et al. [10] found that over 60% of caregivers for children diagnosed with ADHD reported feeling severely burdened. Faden et al. [11] also showed that parents of children with neurodevelopmental disorders in Saudi Arabia exhibited high levels of parental stress, with low levels of quality of life which are exacerbated by increasing severity of the child's symptoms. A more recent study surveyed four regions in Saudi Arabia and explored the negative impact on all domains of the quality of life, especially the environmental one [12].

No previous research has addressed this issue in the Al-Ahsa region. So, this study sought to assess the degree of burden and level of family dysfunction, as well as quality of life among caregivers of children diagnosed with ADHD. Furthermore, to determine the sociodemographic characteristics associated with these problems. 

## Materials and methods

This cross-sectional study was conducted at the Developmental and Behavioral Disorders Center, Maternity and Children Hospital, Al-Ahsa, Saudi Arabia between December 2023 and March 2024. This center is the only governmental health facility that accepts referrals from primary health care centers for the diagnosis and management of ADHD cases. 

Several studies conducted in Saudi Arabia have shown a prevalence of ADHD of 8%. Using Epi-Info software, a sample size of 126 participants was estimated to achieve 80% power, 5% type 1 error, and 95% confidence interval. A systematic random sampling method was used to recruit participants. 

The study included male and female caregivers of children with a confirmed diagnosis of any type of ADHD, who exhibited symptoms before the age of seven, were fluent in Arabic, and agreed to participate in the study. Caregivers of children 17 years of age or older with a serious mental illness such as schizophrenia, addiction, depressive disorder, or autism spectrum disorder were excluded. Caregivers who were unable to give consent were also excluded. 

An online survey created with Google Forms was used to collect data. It consisted of three parts. The first part included sociodemographic data such as gender, age, nationality, education level, marital and employment status, number of children and their relationship to the child, household income per month, health insurance, and the age of the child at the time of ADHD diagnosis. The second part was the Zarit Burden Interview questionnaire to assess the level of burden experienced [13]. The third part was the validated Arabic short version of the World Health Organization Quality of Life instrument (WHOQOL-BREF) to assess the quality of life [14]. This instrument has been widely used in similar contexts and consists of four subdomains that aim to assess QOL in four basic areas: physical, psychological, social, and environmental. There are 26 items in the questionnaire, two of which focus on general health, while the remaining items are distributed as follows: Eight items pertain to the environmental domain, three to the social interactions domain, six to the psychological domain, and seven to the physical domain. Responses to the items are scored on a 5-point Likert scale ranging from 1 to 5, with higher scores indicating more positive responses. The corresponding item scores within each domain were summed after data collection to determine domain scores. These domain scores were then converted to a positive total score of 0-100 [15].

To assess the level of family dysfunction, an Arabic version of the activity (tone), pulse, grimace, appearance, and respiration (APGAR) scale was used to measure the five components of perceived family functioning, including adaptability, participation, growth, affection, and resolution. Each of the five questions had three options (almost never, occasionally, and almost often) and scores ranged from 0 to two. Families received a total score of 0 to 10, with 7-10 representing functional families and ≤ 6 representing dysfunctional families. It is also possible to categorize dysfunctional families as severely (≤ 3) or moderately (4-6) dysfunctional [16]. 

The researchers contacted randomly selected caregivers by telephone. The online survey was distributed to the participants via commonly used social media platforms (WhatsApp and Telegram). 

The study received ethical approval from the Research Ethics Committee of King Fahad Hospital Hofuf (ID:17-E-2023) The consent to participate was signed voluntarily by the participants after they were informed about the objectives of the study. The confidentiality of participants' personal information was maintained by assigning a code number to each participant.

Statistical analysis

The data were collected, reviewed, and then fed to Statistical Package for Social Sciences version 26 (IBM Inc., Armonk, NY, USA). All statistical methods used were two-tailed with an alpha level of 0.05 considering significance if the p value was less than or equal to 0.05. Descriptive analysis was done by prescribing frequency distribution and percentage for study variables including ADHD caregivers’ personal data, degree of burden, degree of function of their families, and domains of life quality according to WHOQOL-BREEF score. The significance of the relation was assessed using the Pearson Chi-square test and exact probability test for small frequency distributions.

## Results

Participants characteristics 

A total of 103 ADHD caregivers were included in the study. Their ages ranged from 14 to 57 years, with a mean of 36.83±7.09 years, and the majority (44, 42.7%) were between 35 and 40 years of age. About half (53, 51.5%) of them were male and most (99, 96.1%) of them were Saudi. The educational level was mainly high school (44, 42.7%) and university (49, 47.6%). Most of the ADHD caregivers (96, 93.2%) were married and seven (6.8%) were divorced. A large proportion of participants were employed (66, 64.1%), followed by homemakers (27, 26.2%). Many of the participants had a monthly income of 6,000-10,000 Saudi Riyal (SR) (28, 27.2%) and 27 (26.2%) had a monthly income of 2,000-5,000 SR. Almost half of the participants (52, 50.5%) were uninsured and the rest (51, 49.5%) had private insurance. Many of the participants (54, 52.4%) were caregivers of one to three children. Most caregivers were either fathers (51, 49.5%) or mothers (48, 46.6%). Two-thirds (68, 66.0%) had help from their spouse (wife or husband). The age of the children with ADHD in this study was mostly (39, 37.9%) seven to 10 years, followed by four to six years (35, 34.0%). The duration of the disorder was mainly (63, 61.2%) one to three years (Table 1).

**Table 1:** Socio-demographic characteristics of ADHD caregivers (n=103) n: number; SD: standard deviation; SR: Saudi Riyal; ADHD: attention deficit hyperactivity disorder

Variables		n	%
Age in years	<30	13	12.6
	30-34	26	25.2
	35-40	44	42.7
	>40	20	19.4
Gender	Male	53	51.5
	Female	50	48.5
Nationality	Saudi	99	96.1
	Non-Saudi	4	3.9
Educational level	University graduate	49	47.6
	High school graduate	44	42.7
	Primary school graduate	10	9.7
Marital status	Married	96	93.2
	Divorced	7	6.8
	Widowed	0	0.0
	Single	0	0.0
Employment status	Unemployed	8	7.8
	Employed	66	64.1
	Housewife	27	26.2
	Retired	2	1.9
Income (SR/month)	<2000	23	22.3
	2000-5000	27	26.2
	6000-10000	28	27.2
	>10000	25	24.3
Health insurance	Uninsured	52	50.5
	Private insurance	51	49.5
Number of persons in care	No	7	6.8
	1-3	54	52.4
	4-6	35	34.0
	>6	7	6.8
Relation to the affected child	Mother	48	46.6
	Father	51	49.5
	Uncle	1	1.0
	Son or daughter	3	2.9
Someone helps you	No	24	23.3
	My husband or wife	68	66.0
	Maid or specialist	2	1.9
	My son or daughter	4	3.9
	Grand father or mother	5	4.9
Age of child with ADHD in years	1-3	9	8.7
	4-6	35	34.0
	7-10	39	37.9
	>10	20	19.4
Time elapsed from first diagnosis of ADHD	Less than 1 year	14	13.6
	1-3 years	63	61.2
	4-6 years	13	12.6
	7-10 years	11	10.7
	>10 years	2	1.9

Degree of burden among participants

Eighty-nine of 103 (86.4%) participants reported varying degrees of caregiver burden. There was a significant association between caregiver marital status and level of strain (p=0.024). Divorced caregivers were mostly (4, 57.1%) severely burdened. However, married caregivers were mostly (40, 41.7%) mildly to moderately burdened. Otherwise, no significant associations were found between other sociodemographic characteristics and burden (all p values >0.05). It was found that caregivers aged <30 years were mostly (6, 46.2%) moderately to severely burdened. However, the rest of the age groups were mostly mildly to moderately burdened. There was a high frequency (25, 47.2%) of mild to moderate strain among men, while there was a high frequency (21, 42.0%) of moderate to severe strain among women. Taking into account the nationality and employment status of the caregivers and the age of the children with ADHD, the majority of the participants were mildly to moderately burdened. The highest percentage of university graduates (20, 40.8%) were moderately to severely burdened, whereas three (30.0%) of primary school graduates were severely burdened. There was a significant association between the caregiver's marital status and level of strain (p = 0.024). Divorced caregivers were mostly (4, 57.1%) severely burdened. However, married caregivers were mostly, (40, 41.7%) mildly to moderately burdened. Regarding monthly income, it was found that caregivers with a monthly income of 5000 SR or less were mostly mildly to moderately burdened. On the other hand, caregivers with a monthly income of 6000 SR or more were mostly moderately to severely burdened. The majority of uninsured caregivers (19, 36.5%) were moderately to severely burdened. However, of the insured caregivers, (23, 45.1%) were mildly to moderately burdened. Caregivers of three or fewer children were moderately to severely burdened. However, those caring for four or more children were mildly to moderately burdened. The majority of caregiving mothers (20, 41.7%) were moderately to severely burdened. On the other hand, fathers (25, 49.0%) were mildly to moderately burdened (Table 2).

**Table 2:** The degree of burden in ADHD caregivers (n=103) n: number; SD: standard deviation; SR: Saudi Riyal; ADHD: attention deficit hyperactivity disorder; FE: Fisher’s exact test; X^2^: Statistic of Pearson’s Chi-square test for independence of observations; * significant at p<0.05; $+ significantly higher than expected if the null hypothesis is true

Variables		Little or no burden	Mild to moderate burden	Moderate to severe burden	Severe burden	P-Value
		n=14	n=41	n=32	n=16	
Age in years	<30	0 (0.0%)	5 (38.5%)	6 (46.2%)	2 (15.4%)	FE, P = 0.875
	30-34	4 (15.4%)	9 (34.6%)	8 (30.8%)	5 (19.2%)	
	35-40	6 (13.6%)	20 (45.5%)	12 (27.3%)	6 (13.6%)	
	>40	4 (20.0%)	7 (35.0%)	6 (30.0%)	3 (15.0%)	
Gender	Male	8 (15.1%)	25 (47.2%)	11 (20.8%)	9 (17.0%)	X^2^ = 5.550, P = 0.135
	Female	6 (12.0%)	16 (32.0%)	21 (42.0%)	7 (14.0%)	
Nationality	Saudi	14 (14.1%)	38 (38.4%)	31 (31.3%)	16 (16.2%)	FE, P = 0.730
	Non-Saudi	0 (0.0%)	3 (75.0%)	1 (25.0%)	0 (0.0%)	
Educational level	University graduate	6 (12.2%)	17 (34.7%)	20 (40.8%)	6 (12.2%)	FE, P = 0.371
	High school graduate	6 (13.6%)	21 (47.7%)	10 (22.7%)	7 (15.9%)	
	Primary school graduate	2 (20.0%)	3 (30.0%)	2 (20.0%)	3 (30.0%)	
Marital status	Married	13 (13.5%)	40 (41.7%)	31 (32.3%)	12 (12.5%)	FE, P = 0.024*
	Divorced	1 (14.3%)	1 (14.3%)	1 (14.3%)	4 (57.1%) $+	
Employment status	Unemployed	2 (25.0%)	4 (50.0%)	1 (12.5%)	1 (12.5%)	FE, P = 0.642
	Employed	8 (12.1%)	24 (36.4%)	23 (34.8%)	11 (16.7%)	
	Housewife	3 (11.1%)	13 (48.1%)	7 (25.9%)	4 (14.8%)	
	Retired	1 (50.0%)	0 (0.0%)	1 (50.0%)	0 (0.0%)	
Income (SR/month)	<2000 SR	1 (4.3%)	13 (56.5%)	8 (34.8%)	1 (4.3%)	FE, P = 0.145
	2000-5000 SR	4 (14.8%)	11 (40.7%)	5 (18.5%)	7 (25.9%)	
	6000-10000 SR	6 (21.4%)	7 (25.0%)	9 (32.1%)	6 (21.4%)	
	>10000	3 (12.0%)	10 (40.0%)	10 (40.0%)	2 (8.0%)	
Health insurance	Uninsured	8 (15.4%)	18 (34.6%)	19 (36.5%)	7 (13.5%)	FE, P = 0.520
	Private insurance	6 (11.8%)	23 (45.1%)	13 (25.5%)	9 (17.6%)	
Number of children in care	No	0 (0.0%)	3 (42.9%)	3 (42.9%)	1 (14.3%)	FE, P = 0.749
	1-3	7 (13.0%)	19 (35.2%)	20 (37.0%)	8 (14.8%)	
	4-6	5 (14.3%)	17 (48.6%)	7 (20.0%)	6 (17.1%)	
	>6	2 (28.6%)	2 (28.6%)	2 (28.6%)	1 (14.3%)	
relation to the affected child	Mother	7 (14.6%)	14 (29.2%)	20 (41.7%)	7 (14.6%)	FE, P = 0.219
	Father	7 (13.7%)	25 (49.0%)	10 (19.6%)	9 (17.6%)	
	Uncle	0 (0.0%)	1 (100.0%)	0 (0.0%)	0 (0.0%)	
	Son or daughter	0 (0.0%)	1 (33.3%)	2 (66.7%)	0 (0.0%)	
Someone helps you	No	2 (8.3%)	5 (20.8%)	11 (45.8%)	6 (25.0%)	FE, P = 0.220
	My husband or wife	9 (13.2%)	32 (47.1%)	17 (25.0%)	10 (14.7%)	
	Maid or specialist	0 (0.0%)	1 (50.0%)	1 (50.0%)	0 (0.0%)	
	My son or daughter	2 (50.0%)	1 (25.0%)	1 (25.0%)	0 (0.0%)	
	Grand father or mother	1 (20.0%)	2 (40.0%)	2 (40.0%)	0 (0.0%)	
Age of child with ADHD	1-3	0 (0.0%)	5 (55.6%)	4 (44.4%)	0 (0.0%)	FE, P = 0.181
	4-6	4 (11.4%)	16 (45.7%)	12 (34.3%)	3 (8.6%)	
	7-10	9 (23.1%)	14 (35.9%)	8 (20.5%)	8 (20.5%)	
	>10	1 (5.0%)	6 (30.0%)	8 (40.0%)	5 (25.0%)	
Time elapsed from first diagnosis of ADHD	Less than 1 year	2 (14.3%)	8 (57.1%)	3 (21.4%)	1 (7.1%)	FE, P = 0.265
	1-3 years	8 (12.7%)	26 (41.3%)	22 (34.9%)	7 (11.1%)	
	4-6 years	3 (23.1%)	3 (23.1%)	3 (23.1%)	4 (30.8%)	
	7-10 years	1 (9.1%)	4 (36.4%)	2 (18.2%)	4 (36.4%)	
	>10 years	0 (0.0%)	0 (0.0%)	2 (100.0%)	0 (0.0%)	

Degree of dysfunction of the families of the ADHD caregivers

Ninety of 103 participants (87.4%) reported having dysfunctional families, and the majority of them (60, 60.1%) reported moderate family dysfunction. For most of the bio-demographic data examined, most caregivers found their families to be moderately dysfunctional. The majority of retired caregivers who received help from a maid, specialist, son or daughter, or who had been caring for ADHD patients for more than 10 years found their families to be severely dysfunctional, with no significant associations. There was an association between the caregiver's marital status and level of dysfunction, but it did not reach the level of significance (p=0.061). Fifty-nine (61.5%) of married caregivers and three (42.9%) of divorced caregivers reported that their families were moderately dysfunctional (Table 3).

**Table 3:** The degree of dysfunction of the families of ADHD caregivers (n=103) n: number; SD: standard deviation; SR: Saudi Riyal; ADHD: attention deficit hyperactivity disorder; FE: Fisher’s exact test; X^2^: Statistic of Pearson’s Chi-square test for independence of observations

Variables		Functional family	Moderately dysfunctional family	Severely dysfunctional family	P-Value Pearson X2 test
		n=13	n=62	n=28	
Age in years	<30	3 (23.1%)	6 (46.2%)	4 (30.8%)	FE, P = 0.769
	30-34	3 (11.5%)	15 (57.7%)	8 (30.8%)	
	35-40	6 (13.6%)	28 (63.6%)	10 (22.7%)	
	>40	1 (5.0%)	13 (65.0%)	6 (30.0%)	
Gender	Male	6 (11.3%)	34 (64.2%)	13 (24.5%)	X^2^ = 0.714, P = 0.700
	Female	7 (14.0%)	28 (56.0%)	15 (30.0%)	
Nationality	Saudi	13 (13.1%)	59 (59.6%)	27 (27.3%)	FE, P = 1.000
	Non-Saudi	0 (0.0%)	3 (75.0%)	1 (25.0%)	
Educational level	University graduate	5 (10.2%)	25 (51.0%)	19 (38.8%)	FE, P = 0.083
	High school graduate	7 (15.9%)	31 (70.5%)	6 (13.6%)	
	Primary school graduate	1 (10.0%)	6 (60.0%)	3 (30.0%)	
Marital status	Married	10 (10.4%)	59 (61.5%)	27 (28.1%)	FE, P = 0.061
	Divorced	3 (42.9%)	3 (42.9%)	1 (14.3%)	
Employment status	Unemployed	0 (0.0%)	6 (75.0%)	2 (25.0%)	FE, P = 0.778
	Employed	11 (16.7%)	38 (57.6%)	17 (25.8%)	
	Housewife	2 (7.4%)	17 (63.0%)	8 (29.6%)	
	Retired	0 (0.0%)	1 (50.0%)	1 (50.0%)	
Income (SR/month)	<2000 SR	2 (8.7%)	15 (65.2%)	6 (26.1%)	FE, P = 0.356
	2000-5000 SR	3 (11.1%)	17 (63.0%)	7 (25.9%)	
	6000-10000 SR	2 (7.1%)	20 (71.4%)	6 (21.4%)	
	>10000	6 (24.0%)	10 (40.0%)	9 (36.0%)	
Health insurance	Uninsured	7 (13.5%)	31 (59.6%)	14 (26.9%)	X^2^ = 0.067, P= 0.967
	Private insurance	6 (11.8%)	31 (60.8%)	14 (27.5%)	
Persons in care	No	1 (14.3%)	3 (42.9%)	3 (42.9%)	FE, P = 0.913
	1-3	8 (14.8%)	32 (59.3%)	14 (25.9%)	
	4-6	4 (11.4%)	22 (62.9%)	9 (25.7%)	
	>6	0 (0.0%)	5 (71.4%)	2 (28.6%)	
Relation to the affected child	Mother	8 (16.7%)	26 (54.2%)	14 (29.2%)	FE, P = 0.263
	Father	4 (7.8%)	34 (66.7%)	13 (25.5%)	
	Uncle	0 (0.0%)	0 (0.0%)	1 (100.0%)	
	Son or daughter	1 (33.3%)	2 (66.7%)	0 (0.0%)	
Someone helps you	No	5 (20.8%)	14 (58.3%)	5 (20.8%)	FE, P = 0.423
	My husband or wife	7 (10.3%)	43 (63.2%)	18 (26.5%)	
	Maid or specialist	0 (0.0%)	1 (50.0%)	1 (50.0%)	
	My son or daughter	0 (0.0%)	1 (25.0%)	3 (75.0%)	
	Grand father or mother	1 (20.0%)	3 (60.0%)	1 (20.0%)	
Age of child with ADHD in years	1-3	1 (11.1%)	5 (55.6%)	3 (33.3%)	FE, P = 0.560
	4-6	6 (17.1%)	21 (60.0%)	8 (22.9%)	
	7-10	3 (7.7%)	27 (69.2%)	9 (23.1%)	
	>10	3 (15.0%)	9 (45.0%)	8 (40.0%)	
Time elapsed from first diagnosis of ADHD	Less than 1 year	3 (21.4%)	7 (50.0%)	4 (28.6%)	FE, P = 0.243
	1-3 years	6 (9.5%)	40 (63.5%)	17 (27.0%)	
	4-6 years	1 (7.7%)	10 (76.9%)	2 (15.4%)	
	7-10 years	3 (27.3%)	5 (45.5%)	3 (27.3%)	
	>10 years	0 (0.0%)	0 (0.0%)	2 (100.0%)	

Domains of quality of life in ADHD caregivers

In terms of age, caregivers under 30 years of age had the lowest mean scores in all domains, while those over 40 years of age had the highest mean scores in the psychological domains. Compared to men, women had lower mean scores for the total and the four QOL domains. University graduate caregivers had the lowest mean scores on the total, physical, and social QOL domains, while primary school graduates had higher mean scores on all QOL domains except the environmental domain. Non-Saudi caregivers had higher scores on all QOL domains except psychological and social domains, which were higher among Saudi caregivers. Divorced caregivers had lower scores on all QOL domains compared to married caregivers. Employed caregivers had the lowest mean scores for all QOL domains, but retired caregivers had the highest mean scores for all QOL domains. Regarding the monthly household income, higher scores were found for the environmental and social domains of quality of life with an income of less than 2000 SR, for the psychological domain with an income of 2000-5000 SR, and for the physical domain with an income of 6000 SR or more. Uninsured caregivers had lower mean scores in most QOL domains. Caregivers helped by grandfather or mother had lower mean scores in all domains of quality of life compared to those helped by son or daughter. There were no statistically significant differences in total and subdomain QOL scores across sociodemographic factors (all p values >0.05) (Table 4).

**Table 4:** Comparison of the WHOQOL-BREEF mean scores in the domains according to the Bio-demographic characteristics of ADHD caregivers SD: standard deviation; SR: Saudi Riyal; ADHD: attention deficit hyperactivity disorder; *: significant at p<0.05

Variables		Overall quality of life and general health Mean±SD	Domains			
			1 (Physical health) Mean±SD	2 (Psychological health) Mean±SD	3 (Environmental health) Mean±SD	4 (Social relationship) Mean±SD
Total		65.78±23.35	59.67±20.39	58.90±14.91	54.92±15.24	60.68±23.58
Age in years	<30	51.92± 31.81	45.60±22.64	48.40±19.21	48.32±16.47	44.87±28.98
	30-34	69.23±22.98	67.86±20.58	60.10±17.25	58.89±20.76	66.03±24.82
	35-40	68.47±19.72	58.36±18.01	60.13±12.07	54.62±11.88	61.74±22.68
	>40	64.38±23.39	61.07±19.56	61.46±12.31	54.69±11.71	61.67±16.31
	P Value	0.120	0.012*	0.055	0.239	0.060
Gender	Male	69.10±22.41	62.67±20.78	60.85±14.02	54.19±14.82	63.68±22.23
	Female	62.25±24.02	56.50±19.66	56.83±15.68	55.69±15.78	57.50±24.76
	P Value	0.137	0.125	0.173	0.620	0.185
Nationality	Saudi	65.40±23.48	59.60±20.16	59.09±15.07	54.89±15.11	61.03±23.02
	Non-Saudi	75.00±20.41	61.61±29.22	54.17±10.21	55.47±20.79	52.08±38.71
	P Value	0.423	0.848	0.520	0.941	0.460
Educational level	University graduate	62.76±24.54	59.40±21.14	59.61±15.87	56.19±15.47	60.03±24.77
	High school graduate	68.47±22.31	59.58±19.53	57.01±14.12	53.62±15.95	60.23±22.65
	Primary school graduate	68.75±22.24	61.43±22.39	63.75±13.33	54.38±11.04	65.83±23.39
	P Value	0.461	0.960	0.395	0.719	0.770
Social status	Married	66.41±23.11	60.68±20.31	59.55±14.63	55.83±14.91	62.07±23.00
	Divorced	57.14±26.86	45.92±17.30	50.00±17.01	42.41±15.29	41.67±25.00
	P Value	0.313	0.064	0.102	0.024*	0.026*
Employment status	Unemployed	62.50±18.90	60.27±25.29	59.38±11.30	57.81±12.72	62.50±17.82
	Employed	62.88±25.09	59.52±20.65	58.02±15.13	53.17±16.39	58.59±24.93
	Housewife	72.22±18.78	59.79±18.89	60.49±15.56	57.75±13.13	63.89±22.17
	Retired	87.50±17.68	60.71±30.30	64.58±20.62	62.50±4.42	79.17±5.89
	P Value	0.175	0.999	0.844	0.465	0.519
Income (SR/month)	<2000 SR	68.48±20.25	59.63±19.28	59.60±13.56	57.61±12.60	67.39±19.93
	2000-5000 SR	66.67±24.02	58.60±21.32	60.03±18.24	53.36±17.25	57.10±24.21
	6000-10000 SR	65.18±24.38	59.31±20.23	56.55±13.00	53.68±14.51	59.23±22.49
	>10000	63.00±25.12	61.29±21.64	59.67±14.72	55.50±16.44	60.00±27.11
	P Value	0.872	0.972	0.813	0.753	0.461
Health insurance	Uninsured	61.54±25.47	60.23±22.02	58.41±15.43	53.49±14.96	61.70±23.47
	Private insurance	70.10±20.32	59.10±18.78	59.40±14.50	56.37±15.52	59.64±23.88
	P Value	0.063	0.780	0.740	0.339	0.660
Persons in care	No	58.93±26.73	54.59±15.53	53.57±20.75	55.36±8.41	55.95±21.36
	1-3	67.13±23.45	57.34±22.12	57.95±15.06	55.61±17.15	60.65±25.97
	4-6	66.43±22.02	64.18±18.30	60.71±13.64	53.75±14.20	60.00±21.66
	>6	58.93±28.61	60.20±20.14	62.50±14.63	54.91±11.10	69.05±16.47
	P Value	0.708	0.419	0.570	0.957	0.760
Relation to the affected child	Mother	62.50±24.73	57.51±20.14	57.12±16.24	55.86±15.76	57.64±24.06
	Father	68.87±22.27	62.32±20.71	61.60±13.13	54.66±14.97	63.89±22.83
	Uncle	50.00±0.00	25.00±0.00	41.67±0.00	31.25±0.00	16.67±0.00
	Son or daughter	70.83±19.09	60.71±7.14	47.22±14.63	52.08±9.55	69.44±12.73
	P Value	0.492	0.232	0.136	0.443	0.129
Someone helps you	No	59.90±23.88	51.64±16.14	54.86±18.21	51.17±18.36	54.86±26.91
	My husband or wife	68.57±22.39	62.87±20.68	60.42±12.73	56.30±13.80	63.97±22.60
	Maid or specialist	68.75±8.84	64.29±5.05	56.25±2.95	56.25±4.42	66.67±0.00
	My son or daughter	78.13±21.35	65.18±24.46	72.92±12.03	61.72±5.92	58.33±9.62
	Grand father or mother	45.00±28.78	48.57±26.92	47.50±20.96	48.13±23.24	43.33±24.58
	P Value	0.105	0.120	0.058	0.438	0.227
Age of child with ADHD in years	1-3	73.61±13.18	58.33±14.62	62.04±14.65	58.33±7.81	73.15±13.68
	4-6	68.57±25.25	63.37±20.60	60.00±15.43	57.86±16.58	61.67±26.45
	7-10	66.03±26.12	60.53±22.69	58.97±16.41	51.04±15.96	58.97±24.44
	>10	56.88±14.89	52.14±16.28	55.42±10.91	55.78±12.92	56.67±19.04
	P Value	0.220	0.265	0.647	0.228	0.343
Time elapsed from first diagnosis of ADHD	Less than 1 Year	65.18±15.64	59.95±14.37	60.42±12.09	55.13±11.86	63.10±19.53
	1-3 years	66.47±25.48	61.73±20.92	58.27±15.38	54.81±15.11	61.11±25.18
	4-6 years	70.19±22.56	56.87±26.22	64.10±16.10	56.73±14.37	59.62±21.20
	7-10 years	57.95±21.85	53.25±17.46	55.30±15.38	53.13±22.14	56.06±24.18
	> 10 years	62.50±17.68	46.43±10.10	54.17±5.89	54.69±15.47	62.50±29.46
	P Value	0.779	0.594	0.620	0.988	0.962

## Discussion

Attention deficit hyperactivity disorder is a serious medical and public health concern because of its negative impact on the family, which has significant societal implications. The issue of quality of life has become increasingly important in the lives of parents of children with special disorders in recent years [17]. Given the lack of previous regional research on this topic, this study aimed to assess the self-reported burden and level of family disruption and quality of life among caregivers of children with ADHD. In addition, the sociodemographic factors associated with these problems were identified.

Caregivers of children with ADHD often face significant financial, social, and emotional challenges in raising their children. In addition to bearing the financial burden of initiating and continuing therapy and acting as an advocate for their child, they also experience other costs, including loss of work and difficulties in their marital relationships. However, there is a lack of evidence-based information on the severity of burden among parents of children with ADHD [18].

In the present study, 89 (86.4%) of participating caregivers reported varying degrees of family burden. The only significant factor influencing the level of burden was marital status; four (57.1%) of divorced caregivers reported being severely burdened. Married caregivers, on the contrary, reported being mild to moderately burdened. Numerous studies have reported on caregiver burden, but the factors associated with it vary widely across studies. This variation is likely due to the use of different psychometric instruments, age differences in the children, study settings, social structures, cultural norms, economic conditions, and accessibility of health care services. 

A study in Nigeria [19] found a high frequency of burden among caregivers of children with ADHD, with only 7.3% (n=5) of them reporting no burden. Severe, moderate, and mild burden was reported by 24.6% (n=17), 36.2% (n=25), and 31.9% (n=22) of caregivers, respectively. The burden was significantly higher in the presence of co-occurring learning disabilities, mental retardation, or oppositional defiant disorder. On the other hand, those with strong social support had much lower caregiver burden. According to an Iranian study, mothers of children with ADHD experienced strain. Mothers of children with combined or severe symptoms also experienced higher levels of strain. Other factors that contributed to the burden included the presence of ADHD in the brother, the degree of cooperation of family members in caring for the child with ADHD, and the birth rank of the child [20]. Another study highlighted that the combined subtype of ADHD is associated with more parental psychopathology and stress than the inattentive subtype [21]. In addition, it has been reported that despite the use of ADHD medication, caregivers of children and adolescents with ADHD reported distress related to work, social activities, family life, and parental worry/stress [18].

In the present study, divorced caregivers were more likely to report severe strain than married caregivers. It appears that cooperation among family members in caregiving reduces perceptions of strain. Research suggests that the complex social dynamics within families and social networks, rather than the individual characteristics of caregivers, are more closely related to feelings of strain [22].

According to the current study, 90 out of 103 participants (87.4%) reported having dysfunctional families, and the majority of caregivers surveyed felt that their families were only moderately dysfunctional (60, 60.1%). The caregiver's marital status was associated with the level of family dysfunction, but it did not reach the level of significance (p=0.061). Thirty-nine caregivers (61.5%) who were married and three (42.9%) who were divorced reported that their families were quite dysfunctional. However, the majority of retired participants, those who had cared for an ADHD patient for more than 10 years, and those who had help from maids, specialists, sons, or daughters reported that their families were extremely dysfunctional, with no significant associations. Azazy et al. [23] found that most families of children with ADHD were dysfunctional. The researchers also reported a significantly higher dysfunctional family among parents aged less than 40 years or living in urban areas, better family function with older age, and higher psychological scores of parents. Another study involving Norwegian fathers and mothers of children with ADHD showed that parents with a child with ADHD had poorer family function than others who did not have a child with ADHD [24]. According to another comparative study, families with children diagnosed with ADHD had significantly higher levels of family dysfunction than families without ADHD, with no significant effect of socioeconomic status on family dysfunction [25]. This suggests that parents of children with ADHD have difficulty with family cohesion and organization [23]. Parents of children with ADHD also have problems with child interaction and dysfunctional parenting styles and experience emotional stress, distress, and exhaustion [26]. Stress levels can rise sharply when the child refuses to comply with routine parental demands, which can negatively impact family dynamics [27]. In families with children with ADHD, simple daily tasks such as completing homework or getting ready for bed can be difficult for families whose children have ADHD. In addition, there is a higher likelihood of marital problems among parents whose children have been diagnosed with ADHD [28]. Parents expressed that they need to learn about communication issues and how to improve their performance while caring for their sick child [29].

The results of the current study showed that all QOL domains were negatively impacted, with the environmental domain experiencing the most disruption. The total and four sub-domains of QOL did not differ significantly between different socio-demographic characteristics.

However, it is worth noting that the lowest mean scores in most QOL categories were reported by caregivers who were under 30 years of age, female, divorced, college graduates, uninsured, employed, and dependent on their grandparents. These findings are consistent with Alhefdhi et al. [12], who recruited participants from four regions in Saudi Arabia and reported a negative impact on all domains of QOL for parents of children with ADHD, particularly the environmental domain. However, they did examine a statistically significant association between being single, unemployed, or having one child and experiencing lower mean scores on the overall QOL. Another study conducted in the Jazan region of Saudi Arabia found that approximately half of the parents of children with ADHD reported poor quality of life. Notably, a higher proportion of female parents than male parents reported poor QOL. They also found no significant associations between parental QOL and employment status, family income, and education level [30].

Research on the quality of life of parents of children with ADHD in Egypt revealed average QOL scores, with most families experiencing dysfunction. There were statistically significant relationships between different domains of parental QOL and education, income, employment, residence, and marital status. Lower QOL scores were recorded among illiterate and only literate parents of children with ADHD who lived in urban areas, were divorced, and had insufficient income, as noted by Azazy et al. [23]. Another Egyptian study reported a significant deterioration in QOL dimensions of caregivers of ADHD children, compared to a control group that included parents of non-ADHD children. The study also documented poor QOL among two-thirds of caregivers of ADHD children [31]. In Bangladesh, parents of children with ADHD reported extremely low QOL scores, with the psychological domain being the most impaired. The study found notable associations between many sociodemographic variables, such as gender, location, family income, number of family members, marital status, occupation, and education level, and the parents' QOL [32]. A study from Taiwan added a consistent positive effect of family support on the health-related quality of life of mothers of children with ADHD [33]. Similar results have been found in studies conducted in several parts of the world, including Tunisia [34], Iran [35], Hong Kong [36], and France [37]. In a similar context, it has been reported that mothers of hyperactive children experience high levels of depression, anxiety, and personality disorders [38].

The documented poor QOL among parents of children with ADHD may be related to their exposure to much higher levels of parenting stress compared to parents of typically developing children. Their children's health status leads to exhaustion, fatigue, stress from inattention, aggressive behavior, and challenges in forming social interactions and peer relationships [29]. It has also been reported that dysfunctional, ineffective parenting, disengagement from parent-child relationships, and child maltreatment negatively affect the physical and mental health of parents [39]. Health education and increasing the level of knowledge of caregivers can significantly reduce stress, improve quality of life, and give them more strength to cope with the burden of the disease [40]. In addition, multilevel interventions that combine effective methods and tools according to the needs of the family have shown positive outcomes for both parents and children, as demonstrated by Pachiti et al. [41].

Overall, our findings show that having an ADHD child can significantly affect parental QOL, independent of sociodemographic characteristics. The lack of a significant effect of family income on caregiver QOL suggests that financial stress may not be the most important determinant of QOL for parents of children with ADHD compared to emotional, social, and parenting stress. This is in line with Ahmed et al. [31] who found significant correlations between the QOL of caregivers of ADHD children and self-liking and self-competence. The above findings underscore the need for a multifaceted strategy that addresses the psychological, structural, and contextual elements that influence parental well-being.

Limitations and strength

There are several limitations to this study that require careful interpretation. There is a possibility of response bias due to the self-report survey approach. Caregivers may underreport or overreport their experiences due to social desirability or embarrassment, particularly regarding their child’s ADHD diagnosis. This bias could lead to an over- or underestimation of the burden and dysfunction levels. In addition, the cross-sectional design of our study restricts the ability to infer causality. While associations between variables like marital status and level of burden were observed in the current study, we cannot conclude that one causes the other. Longitudinal studies would be needed to establish cause-and-effect relationships. Furthermore, there is also the possibility of non-response bias, where caregivers experiencing the most severe burdens may have been less likely to participate in the study. This could result in an underrepresentation of those facing the most significant challenges. Despite these drawbacks, it's important to remember that our research used validated questionnaires, which improved the internal consistency of our conclusions. It is also one of the few studies to assess quality of life in the Al-Ahsa region of Saudi Arabia. This helps to fill a gap in knowledge about the impact of childhood ADHD on parental well-being in the context of Saudi Arabia's distinct social structure. This may help to develop culturally sensitive interventions that will improve parental quality of life and assist Saudi families in managing ADHD. 

## Conclusions

The results of the present study indicate a high prevalence of burden among caregivers of children with ADHD, with the majority perceiving family dysfunction. Marital status was the only significant sociodemographic factor contributing to the level of burden. In addition, all domains of quality of life were negatively affected, particularly the environmental domain, but the sociodemographic variables were not associated with caregiver well-being. Taken together, these data highlight the difficulties faced by parents of children with ADHD and the need for psychosocial interventions designed with the Saudi cultural context in mind. Family therapy and other interventions aimed at improving caregiver-family relationships should also be considered. Improving parental coping and quality of life in families dealing with childhood ADHD may lead to better outcomes for the child.
